# Metabolomics and *16S rRNA* sequencing of human colorectal cancers and adjacent mucosa

**DOI:** 10.1371/journal.pone.0208584

**Published:** 2018-12-21

**Authors:** Mun Fai Loke, Eng Guan Chua, Han Ming Gan, Kumar Thulasi, Jane W. Wanyiri, Iyadorai Thevambiga, Khean Lee Goh, Won Fen Wong, Jamuna Vadivelu

**Affiliations:** 1 Department of Medical Microbiology, Faculty of Medicine, University of Malaya, Kuala Lumpur, Malaysia; 2 Marshall Centre for Infectious Disease Research and Training, School of Biomedical Sciences, University of Western Australia, Perth, Australia; 3 Monash University Malaysia Genomics Facility, School of Science, Monash University Malaysia, Selangor Darul Ehsan, Malaysia; 4 Department of Medicine, Johns Hopkins University School of Medicine, Johns Hopkins Medical Institutions, Baltimore, Maryland, United States of America; 5 Department of Medicine, Faculty of Medicine, University of Malaya, Kuala Lumpur, Malaysia; University of South Alabama Mitchell Cancer Institute, UNITED STATES

## Abstract

Colorectal cancer (CRC) is ranked the third most common cancer in human worldwide. However, the exact mechanisms of CRC are not well established. Furthermore, there may be differences between mechanisms of CRC in the Asian and in the Western populations. In the present study, we utilized a liquid chromatography-mass spectrometry (LC-MS) metabolomic approach supported by the *16S rRNA* next-generation sequencing to investigate the functional and taxonomical differences between paired tumor and unaffected (normal) surgical biopsy tissues from 17 Malaysian patients. Metabolomic differences associated with steroid biosynthesis, terpenoid biosynthesis and bile metabolism could be attributed to microbiome differences between normal and tumor sites. The relative abundances of *Anaerotruncus*, *Intestinimonas* and *Oscillibacter* displayed significant relationships with both steroid biosynthesis and terpenoid and triterpenoid biosynthesis pathways. Metabolites involved in serotonergic synapse/ tryptophan metabolism (Serotonin and 5-Hydroxy-3-indoleacetic acid [5-HIAA]) were only detected in normal tissue samples. On the other hand, S-Adenosyl-L-homocysteine (SAH), a metabolite involves in methionine metabolism and methylation, was frequently increased in tumor relative to normal tissues. In conclusion, this study suggests that local microbiome dysbiosis may contribute to functional changes at the cancer sites. Results from the current study also contributed to the list of metabolites that are found to differ between normal and tumor sites in CRC and supported our quest for understanding the mechanisms of carcinogenesis.

## Introduction

Colorectal cancer (CRC) is the third most commonly occurring cancer in men and the second most commonly occurring cancer in women worldwide; almost 55 percent of CRC cases occur in more developed regions [1]. The estimated 2012 age-standardized incidence rates (per 100 000 population) in Southeast Asia region are 8.9 and 6.3 cases in men and women, respectively [1]. The estimated 2012 age-standardized mortality rates (per 100 000 population) in the region are 6.3 and 4.4 cases in men and women, respectively [1]. More than 90% of colorectal carcinomas are adenocarcinomas originating from epithelial cells of the colorectal mucosa; most colorectal adenocarcinomas (~70%) are diagnosed as moderately differentiated while well and poorly differentiated carcinomas account for only 10% and 20%, respectively [2].

The right and left colons, which are developed distinctively from the embryological mid-gut and hindgut, and are joined at the proximal 2/3 and distal 1/3 of the transverse colon. Hence, anatomically, the blood supply, innervation, lymphatic drainage, and lumen environment are different between right (ascending, proximal through the hepatic flexure) and left (descending, distal to the hepatic flexure) colons [3]. Primary CRCs are more frequent on the left colon but a tendency for right-shift of the primary CRC site has been noted in recent years [4]. Patients with right-sided CRC were older, mostly female, more frequently presenting with advanced tumor stages with larger tumor sizes, more often poorly differentiated tumors, and different molecular biological tumor patterns [5]. Drewes et al. confirmed in the Malaysian cohort (designated as MAL2) that invasive biofilms, *Bacteroides fragilis* and several oral pathogens are enriched in CRC tissues [6].

A deeper understanding on colonic metabolism is needed to identify cancer-related biomarkers to elucidate the cancerous cell progression in CRCs. In addition to genomics and proteomics, which are eminent tools for cancer studies, metabolomics is emerging as a tool to discover biomarkers and unravel pathological processes [7]. The metabolic fingerprints of specific cellular processes and/or low-weighted molecule profiles are prioritized in metabolomics. Valuable scientific insight including in toxicity studies has occurred through metabolomic data generated through nuclear magnetic resonance (NMR), gas chromatography-mass spectrometry (GC-MS), capillary electrophoresis-mass spectrometry (CE-MS), and more recently, liquid chromatography-mass spectrometry (LC-MS) [8,9]. LC-MS is suited for the analysis of chemically diverse low molecular weight compounds produced during human metabolic processes [9]. For this current study, LC-Quadrupole Time-of-Flight (Q-TOF) MS was chosen to explore potential metabolomic biomarkers distinguishing healthy from cancerous tissues in the human colon. This technology has been proven to significantly improve mass accuracy and resolution, besides displaying high sensitivity, good isotopic fidelity, reproducibility of retention time, and optimization of data acquisition.

In this study, 17 paired surgical biopsy tissues that are subset of the MAL2 cohort [6] were included. Biofilm quantification and *16S rRNA* sequencing have been performed and *16S rRNA* data were analyzed using Resphera Insight, a clinical-grade proprietary analysis protocol [6]. Thus, in the current study, metabolomic analysis of paired normal–tumor tissues from patients with colorectal cancer to identify differences metabolomic profiles between cancerous and adjacent non-cancerous tissues obtained from the colon. In addition, *16S rRNA* data were reanalyzed to discover possible association between metabolomic differences and microbiome aberrations. A hallmark of cancer is metabolic reprogramming. However, the underlying mechanism of metabolic reprogramming in cancer is complex and not well understood. Since many lifestyle-related factors have been linked to CRC and limited studies of this nature have been conducted in the Southeast Asian population, this study was expected to present a different perspective on metabolomics and the microbiome of CRC in this population.

## Materials and Methods

### Ethical statement

The University of Malaya Medical Centre (UMMC, Kuala Lumpur, Malaysia) Medical Ethics Committee (Ref. No. 1066.38) and the Johns Hopkins Institutional Review Board approved this study. All biopsy tissues were collected after obtaining informed and written consents from the patients.

### Sample collection

Samples were collected consecutively from 2013 to 2014. The details of colon tissues collection method though a standard mechanical bowel preparation have been previously described [6,10]. Exclusion criteria include those individuals who have received pre-operative radiation, chemotherapy treatment or had a past history of CRC or inflammatory bowel disease. Standard pre-operative intravenous antibiotics (cefotetan, clindamycin/ gentamicin or cefoperazone/ metronidazole) were administered in all cases and none of the patients received any pre-operative oral antibiotics. Tissue from the proximal colon through the hepatic flexure is considered as right (ascending) colon, whereas distal to the hepatic flexure as left (descending) colon. During surgery, excess colon tumor (adenomas and cancers) and paired tumor-free (herein referred to as “normal”) tissues were collected for analysis. Detailed demography and characteristics of the colonic tissue of the study patients are as summarized in Table 1. Statistical significance of age, racial and gender distributions between patients presenting with left and right colorectal tumors were assessed using one-way ANOVA (IBM SPSS Statistics version 22.0) with *p*-value < 0.05 considered significant.

**Table 1:** Demographic information and characteristics of colonic tissues.

Patient ID	Gender	Race	Age (During surgery)	Type	Location	Anatomic Stage (TNM)	Biofilm (Normal)	Biofilm (CRC)
**S035**	Male	Indian	74	Moderately Differentiated Adenocarcinoma	Left	IIIB	No	No
**S036**	Female	Chinese	49	Moderately Differentiated Adenocarcinoma	Left	IVB	No	No
**S037**	Female	Chinese	57	Moderately Differentiated Adenocarcinoma	Left	IIIA	Yes	Yes
**S038**	Male	Malay	49	Moderately Differentiated Adenocarcinoma	Left	IIIB	Yes	Yes
**S039**	Female	Chinese	75	Poorly Differentiated Adenocarcinoma	Right	IIA	No	Yes
**S040**	Female	Chinese	84	Moderately Differentiated Adenocarcinoma	Left	I	Yes	Yes
**S043**	Female	Malay	52	Moderately Differentiated Adenocarcinoma	Left	IIA	No	No
**S044**	Male	Indian	41	Well Differentiated Adenocarcinoma	Right	IVB	No	Yes
**S045**	Female	Chinese	50	Adenocarcinoma	Right	I	Yes	Yes
**S046**	Female	Chinese	52	Adenocarcinoma (Mucinous)	Left	IIA	Yes	Yes
**S048**	Female	Malay	74	Moderately Differentiated Adenocarcinoma	Left	IVA	No	No
**S051**	Female	Indian	63	Moderately Differentiated Adenocarcinoma	Right	IIA	Yes	Yes
**S053**	Male	Chinese	54	Moderately Differentiated Adenocarcinoma	Left	I	No	No
**S056**	Male	Indian	76	Moderately Differentiated Adenocarcinoma	Right	IV	Yes	Yes
**S057**	Male	Chinese	73	Moderately Differentiated Adenocarcinoma	Left	IIIB	No	No
**S058**	Female	Chinese	67	Moderately Differentiated Adenocarcinoma	Left	IIA	No	No
**S060**	Male	Chinese	72	Moderately Differentiated Adenocarcinoma	Right	IIA	Yes	Yes

Samples in this Table are from the MAL2 cohort first reported in Drewes et al [5]. Raw sequences from the MAL2 cohort have been deposited in the NCBI SRA repository (BioProject accession no. PRJNA325650). Primary sequencing data are available upon request.

### Metabolite extraction

The tissue, disposable pestle and 1.5 ml-centrifuge tube in liquid nitrogen were chilled in liquid nitrogen. The tissue was pulverized in the presence of liquid nitrogen to fine powder. Metabolites were extracted from tissue samples by the Bligh and Dyer extraction method [11]. Briefly, 100 μl of chloroform (HPLC grade; Friendemann Schmidt Chemical, Australia) and 200 μl of methanol (HPLC grade; Friendemann Schmidt Chemical, Australia) were added to the fine powder and resuspended by vigorous vortexing. The mixture was stored at room temperature for 30 min. Subsequently, 100 μl of chloroform and 100 μl of water were added and mixed. The tube was centrifuged at 12,000 xg for 10 min. The biphasic solutions were separated into two separate tubes without disturbing the protein precipitate at the interface. The samples were vacuum concentrated to dryness in a Refrigerated CentriVap concentrator (Labconco, USA) at 4°C. The samples were reconstituted with 20 μl of mobile phase (95% water:5% ACN), vortexed and centrifuged at 12,000 xg for 10 minutes at 4°C.

### Untargeted metabolomics by LC/MS

The samples were analyzed on an Agilent 1260 Infinity-6540 UHD Accurate-Mass Quadrupole-Time-of-Flight (Q-TOF) LC/MS system coupled with Dual Agilent Jet Stream Electrospray Ionization source (Agilent Technologies, USA). The injection volume was 3 μl of sample and separation was using a Zorbax Eclipse plus C-18 Rapid Resolution High Throughput (RRHT) 2.1x 100mm 1.8 μm column (Agilent Technologies, USA). The separation was performed at a flow rate of 0.45 mL/min with linear gradient program. Mobile phase A composed of 0.1% formic acid in Milli-Q water and mobile phase B composed of 0.1% formic acid in acetonitrile (HPLC grade; Friendemann Schmidt Chemical, Australia) The gradient program was set as follows: t = 0 min, 5% B; t = 2 min, 5% B; t = 15 min, 98% B; t = 18min, 98%; t = 20 min, 5% B and the final stop time, t = 25 min, 5% B. For positive ionization mode, two reference masses of (i) 121.0509 m/z and (ii) 922.0098 m/z were measured continuously while for negative ionization mode, the reference masses were (i) 112.9855 and (ii) 1033.9881. Reference mass correction was enabled. The gas temperature was maintained at 300°C, drying gas flow was set at the rate of 8 L/min, sheath gas temperature and sheath gas flow at 350°C and 11 L/min respectively. The capillary voltage was 3500 V. The nebulizer pressure was set at 35 psi. The MassHunter Workstation software B.05.01 (Agilent Technologies, USA) was applied for instrument control and data acquisition. The data was analyzed using the Mass Profiler Professional software version 12.6.1 (Agilent Technologies, USA and Strand Life Sciences, USA). We compared the relative abundances between paired tumor and adjacent normal tissue samples using the non-parametric two-sided Wilcoxon signed rank test. Differences were considered significant with p-value < 0.05. The procedure has been deposited at protocols.io: http://dx.doi.org/10.17504/protocols.io.u4ceysw.

### DNA extraction

Genomic DNA was isolated using the MasterPure DNA Purification Kit (Epicentre/Illumina). The primers, S-D-Bact-0341-b-S-17 forward (5′-NNNNCCTACGGGNGGCWGCAG-3′) and S-D-Bact-0785-a-A-21 reverse (5′-GACTACHVGGGTATCTAATCC-3′), including Illumina-compatible adapters, were used to amplify the V3-V4 region of the *16S rRNA* gene [6].

### Analysis of *16S rRNA* amplicon sequence data

The 16S sequencing data were quality-trimmed using Sickle (github.com/najoshi/sickle) using the following parameters: -q 20 –l 200. Merging of overlapping paired-end sequences was performed using MeFit with default parameters [12]. Filtering of chimeric sequences, de novo clustering of *16S rRNA* sequences into Operational Taxonomic Units (OTUs) at 97% similarity threshold and removal of singleton OTUs were conducted using Micca (version 1.7.0) [13]. Taxonomic assignment of the representative OTUs was performed using the Bayesian LCA-based taxonomic classification method with a 1e-100 cut-off e-value and 100 bootstrap replications, against NCBI 16S microbial database [14,15]. Taxonomic assignment at each level was accepted only with a minimum confidence score of 80. Multiple sequence alignment of the OTU representative sequences was performed using PASTA [16]. A phylogenetic tree was constructed using FastTree (version 2.1.8) under the GTR+CAT model [17].

The rarefaction depth value was set at 72290 and subsequent computation of alpha and beta diversities was performed using QIIME (version 1.9.1) [18]. Briefly, alpha diversity was evaluated based on the following metrics: observed species and Shannon diversity index. Non-parametric two-sample t-test was used to compare the alpha diversity metrics between the normal and tumor samples (i.e. using Monte Carlo permutations to calculate the p-value). Principle coordinates analysis (PCoA) using unweighted UniFrac distance metric was performed to visualize separation of samples. Non-parametric statistical analysis of the distance metric was performed using ANOSIM with 1000 permutations. Graphs were generated using both phyloseq R package and PhyloToAST software [19,20].

Functional profiling based on KEGG pathways was conducted using Piphillin [21]. To generate the microbial community correlation networks, the Kendall’s tau correlation coefficients between rarefied abundances of different bacterial genera were calculated using the SparCC software [22]. The statistical significance of each pairwise comparison was examined by bootstrapping with 500 iterations. Only negative and positive correlations of values ≤ -0.7 and ≥ 0.7, respectively, and with pseudo *p*-values of less than 0.01, were considered. The networks were visualized using Cytoscape software 3.6.1 [23].

Pearson’s correlation analysis of bacterial genera against KEGG pathways, and bacterial genera against metabolites, was performed on rarefied abundance data using microbiomeSeq R package (https://github.com/umerijaz/microbiomeSeq.git). Correlations with *p*-values < 0.01 were considered significant.

Genus-level OTUs, KEGG functional pathways and metabolomics compounds with a minimum relative abundance of 0.001% and a detection frequency of at least 25% in all samples, were compared between matched normal and tumor samples by using two-sided Wilcoxon signed rank test. Data with *p*-values < 0.01 were considered significant.

## Results

Among the 17 patients, 11 had left-sided CRC and 6 had right-sided CRC (Table 1). The mean age of patients with left-sided CRC at time of surgery was 60.9 (95% CI: 54.2–67.6) years old while the mean age of patients with right-sided CRC was 62.8 (95% CI: 47.6–78.1) years old. The difference between ages of patients at time of surgery was not statistically significant (one-way ANOVA, *p*-value ≥ 0.05). In the group of left-sided CRC patients, 4 were male and 7 were female. The right-sided group comprised of 3 male and 3 female patients. Racial distribution within the left-sided CRC group was 7 Chinese (64%), 3 Malay (27%) and 1 Indian (9%). On the other hand, right-sided CRC group comprised of 3 Chinese (50%) and 3 Indian (50%). The difference between racial distributions of the two groups was also not statistically significant (one-way ANOVA, *p*-value ≥ 0.05). It is noted that 4 out of 11 left-sided tumors were biofilm positive (36.4%), whereas the six right-sided tumors were biofilm positive (100%). However, 6/8 subjects with biofilm at the site of tumor also had biofilm at adjacent unaffected site (75%).

### Metabolomics

In total, 708 compounds present in more than one sample were annotated (S1 Table). Among these, only 158 compounds had minimum relative abundance of 0.001% and detection frequency of at least 25% in all samples. Table 2 shows 36 compounds found in Kyoto Encyclopedia of Genes and Genomes (KEGG)/ LIPID MAPS Proteome (LMP)/ NIH Human Microbiome Project (HMP) databases and were detected in normal tissues only. In addition, 14 compounds were significantly more frequently found to be higher in normal mucosa than paired tumor tissues (Table 3). Diketospirilloxanthin, which is involve in carotenoid biosynthesis, was detected only in normal tissues (2-tailed Fisher’s exact test, *p*-value = 0.007). 5-hydroxyindoleacetic acid (5-HIAA) and serotonin, which are involved in tryptophan metabolism and serotonergic synapses, were also found only in normal tissues (Fisher’s exact test, *p*-value = 0.044). Serotonin, together with glycocholic acid and cortolone-3-glucuronide, found in normal tissues only (Fisher’s exact test, *p*-value = 0.044), are involved in bile secretion. Furthermore, the level of spermidine, which also plays a role in bile secretion, was significantly more frequently increased in normal mucosa compared to paired tumor tissues (Wilcoxon signed rank test, *p*-value = 0.021) (Table 3). Other metabolites that were present in normal tissues only include PE-Cer(d14:1(4E)/23:0) (Fisher’s exact test, *p*-value = 0.018), 26-O-beta-D-glucopyranosyl-3beta,26-dihydroxy-25(R)-furosta-5,20(22)-dien-3-O-alpha-L-rhamnopyranosyl(1–2)-beta-D-glucopyranoside (*p*-value = 0.018), ganglioside GA1 (d18:1/9Z-18:1) (*p*-value = 0.044) and Pro Arg Ile (*p*-value = 0.044). Phenanthrene-4,5-dicarboylate and m-coumaric acid have roles in aromatic compound degradation. Notably, many of these compounds are listed in the KEGG database to be implicated in diverse metabolic functions.

**Table 2:** Metabolites detected only in normal adjacent mucosa. N, normal adjacent mucosal tissue; L, left-sided colon; R, right-sided colon.

Compound	Frequency (n = 17)	KEGG ID	LMP ID	Description
**Dolichyl beta-D-glucosyl phosphate**	2	C01246	LMPR03080014	Metabolic pathways; N-Glycan biosynthesis
**Diketospirilloxanthin/ 2,2'-Diketospirilloxanthin**	7	C15885	LMPR01070154	Metabolic pathways; Carotenoid biosynthesis
**Glycocholic acid**	1	C01921	LMST05030001	Metabolic pathways, Bile secretion; Cholesterol metabolism, Secondary bile acid biosynthesis, Primary bile acid biosynthesis
**Cortolone-3-glucuronide**	2	C03033	-	Metabolic pathways, Bile secretion; Pentose and glucuronate interconversions
**Coproporphyrin**	1	C03263	-	Metabolic pathways, Biosynthesis of secondary metabolites, Porphyrin and chlorophyll metabolism
**Pyropheophorbide a**	1	C18064	-	Biosynthesis of secondary metabolites, Porphyrin and chlorophyll metabolism
**5-HIAA / 5-Hydroxy-3-indoleacetic acid**	2	C05635	-	Metabolic pathways, Tryptophan metabolism, Serotonergic synapse
**Serotonin**	4	C00780	-	Alkaloids; Metabolic pathways, Bile secretion, Tryptophan metabolism, Serotonergic synapse; Taste transduction, Synaptic vesicle cycle, Neuroactive ligand-receptor interaction, Gap junction, cAMP signaling pathway, Inflammatory mediator regulation of TRP channels
**Tetrahydrofolic acid**	1	C00101	-	Metabolic pathways, Microbial metabolism in diverse environments; Aminoacyl-tRNA biosynthesis, One carbon pool by folate, Antifolate resistance, Methane metabolism, Carbon fixation pathways in prokaryotes, Folate biosynthesis, Carbon metabolism, Glycine, serine and threonine metabolism
**Phenanthrene-4,5-dicarboxylate**	2	C18252	-	Microbial metabolism in diverse environments; Polycyclic aromatic hydrocarbon degradation
**trans,trans-Farnesol**	4	C01126	LMPR0103010001	Terpenoids/Sesquiterpenoids (C15); Biosynthesis of secondary metabolites; Insect hormone biosynthesis, Sesquiterpenoid and triterpenoid biosynthesis, Terpenoid backbone biosynthesis, Biosynthesis of antibiotics
**Adrenosterone**	1	C05285	-	Steroid hormone biosynthesis
**2-Methoxyestrone 3-sulfate**	3	C08358	LMST05020006	Steroid hormone biosynthesis
**2-Methoxyestrone 3-glucuronide**	1	C11132	LMST05010010	Steroid hormone biosynthesis
**Veratridine**	1	C06544	-	Alkaloids
**Cardiopetalidine**	1	C08667	-	Alkaloids
**makisterone B**	1	C08829	LMST01031017	Terpenoids/Steroids
**Convallamaroside**	1	C08892	-	Terpenoids/Steroids
**Quassin**	1	C08778	LMPR0106110002	Terpenoids/Triterpenoids (C30)
**Cucurbitacin A**	1	C08793	LMST01010103	Terpenoids/Triterpenoids (C30)
**Cucurbitacin B**	1	C08794	LMST01010104	Terpenoids/Triterpenoids (C30)
**Cucurbitacin L**	1	C08802	LMST01010112	Terpenoids/Triterpenoids (C30)
**26-O-beta-D-glucopyranosyl-3beta,26-dihydroxy-25(R)-furosta-5,20(22)-dien-3-O-alpha-L-rhamnopyranosyl(1–2)-beta-D-glucopyranoside**	6	-	LMST01070008	-
**PE-Cer(d14:1(4E)/23:0)**	6	-	LMSP03020010	-
**CDP-DG(12:0/12:0)**	3	C13891	LMGP13010001	-
**Pro Arg Ile**	5	-	-	-
**PIP2(16:0/20:3(5Z,8Z,11Z))**	3	C00626	-	-
**Deoxy-5-methylcytidylate**	4	C03495	-	-
**Ganglioside GA1 (d18:1/9Z-18:1)**	5	C06136	-	-
**Losartan**	2	C07072	-	-
**Probucol**	2	C07373	-	-
**Oxyphencyclimine**	2	C07851	-	-
**Lauryl hydrogen sulfate**	3	C11166	-	-
**Telithromycin**	3	C12009	-	-
**Leucomycin A3**	2	C12662	-	-
**9alpha-Fluoro-11beta,16alpha,17alpha,21-tetrahydroxypregn-4-ene-3,20-dione**	2	C14638	-	-

**Table 3:** Metabolites found to be significantly different in paired normal and tumor tissues by Wilcoxon signed rank test.

Compound	Frequency (increased in normal mucosa) (n = 17)	Frequency (increased in tumor) (n = 17)	KEGG ID	LMP ID	*z*-score	*p*-value	Description
**m-Coumaric acid**	14	3	C12621	-	-3.107	0.002	Microbial metabolism in diverse environments, Degradation of aromatic compounds, Phenylalanine metabolism
**N(alpha)-t-Butoxycarbonyl-L-leucine**	12	2	C04301	-	-3.053	0.002	-
**PA(18:4(6Z,9Z,12Z,15Z)/20:4(5Z,8Z,11Z,14Z))**	2	11	-	LMGP10010446	-2.760	0.006	-
**Formylmethionyl-leucyl-phenylalanine methyl ester**	10	1	C11221	-	-2.756	0.006	-
**Arg Arg Met**	2	11	-	-	-2.691	0.007	-
**Acetyl-maltose**	13	4	C02130	-	-2.380	0.017	-
**Spermidine**	11	6	C00315	-	-2.312	0.021	Metabolic pathways, ABC transporters, Bile secretion, Arginine and proline metabolism, beta-Alanine metabolism, Glutathione metabolism, Phenylpropanoid biosynthesis
**Trp Pro Cys**	11	5	-	-	-2.291	0.022	-
**Indoleacrylic acid**	13	4	-	-	-2.249	0.025	-
**SAH / S-Adenosyl-L-homocysteine**	2	12	C00021	-	-2.222	0.026	Metabolic pathways, Biosynthesis of amino acids, Cysteine and methionine metabolism
**Ranitidine**	8	3	C07233	-	-2.197	0.028	-
**Arg Glu Leu**	8	3	-	-	-2.172	0.030	-
**3-Amino-3-(4-hydroxyphenyl)propanoate**	6	1	C04368	-	-2.059	0.040	Tyrosine metabolism
**Pirbuterol**	7	1	C07807	-	-2.059	0.040	-

Table 4 shows 16 compounds that were detected in cancerous tissues only. Among these, 7 compounds were involved in biosynthesis of antibiotics (Macrolides/ Type II polyketide) (Fisher’s exact test, *p*-value = 0.044). In addition, Amphotericin B, a Type I polyketide antifungal agent, was also found in tumor but not normal tissues. Cinnamyl benzoate was also detected only in tumor tissues (Fisher’s exact test, *p*-value = 0.044). Similarly, only 3 out of 14 compounds found to be differentially present in paired normal-tumor tissues were significantly more frequently elevated in tumor tissues (Table 3). Among these was S-adenosyl-L-homocysteine (SAH) that is involved in cysteine and methionine biosynthesis.

**Table 4:** Metabolites detected only in colorectal tumor. T, colorectal tumor; L, left-sided colon; R, right-sided colon.

Compound	Frequency (n = 17)	KEGG ID	LMP ID	Description
**FAD / Flavin adenine dinucleotide**	1	C00016	-	Metabolic pathways, Biosynthesis of secondary metabolites; Vitamin digestion and absorption, Riboflavin metabolism
**PE(18:3(6Z,9Z,12Z)/22:6(4Z,7Z,10Z,13Z,16Z,19Z))**	1	C00350	-	Metabolic pathways, Biosynthesis of secondary metabolites; Glycosylphosphatidylinositol (GPI)-anchor biosynthesis, Autophagy, Glycerophospholipid metabolism, Pathogenic Escherichia coli infection, Retrograde endocannabinoid signaling, Kaposi’s sarcoma-associated herpesvirus infection
**Presqualene diphosphate**	1	C03428	LMPR0106010003	Biosynthesis of antibiotics, Metabolic pathways, Biosynthesis of secondary metabolites; Sesquiterpenoid and triterpenoid biosynthesis, Carotenoid biosynthesis, Steroid biosynthesis
**6,8a-Seco-6,8a-deoxy-5-oxoavermectin ''2b'' aglycone**	1	C11953	-	Biosynthesis of 12-, 14- and 16-membered macrolides
**Avermectin A1b monosaccharide**	1	C11966	-	Biosynthesis of antibiotics, Biosynthesis of 12-, 14- and 16-membered macrolides
**5-O-beta-D-Mycaminosyltylonolide**	1	C12002	-	Biosynthesis of antibiotics, Biosynthesis of 12-, 14- and 16-membered macrolides
**Tetracenomycin B3**	1	C12369	-	Biosynthesis of antibiotics, Biosynthesis of type II polyketide products
**Stealthin C**	2	C12392	-	Biosynthesis of antibiotics, Biosynthesis of type II polyketide products
**antioside**	2	C08848	LMST01120016	Terpenoids/Steroids
**Sarsaparilloside**	2	C08911	-	Terpenoids/Steroids
**Glaucarubin**	2	C08760	-	Terpenoids/Triterpenoids (C30)
**Amphotericin B**	3	C06573	LMPK06000002	Type I polyketide structures
**Cinnamyl benzoate**	5	-	-	-
**Verapamil**	2	C07188	-	-
**Enalkiren**	2	C07466	-	-
**Xamoterol**	4	C11775	-	-

### Pre- and post-processing 16S data and microbiota composition at phylum level

A total of 5,372,109 reads were generated for 17 pairs of normal-tumor samples. Following quality trimming and merging of overlapping paired-end reads, 5,120,010 sequences were retained, ranging from 82,879 to 245,890 per sample with an average read length of 457.6 ± 3.3 bp (S2 Table). Of 1144 OTUs acquired by *de novo clustering* of the merged overlapping paired-end sequences, 662 were taxonomically assigned down to genus level with an 80% confidence threshold.

**Fig 1:**
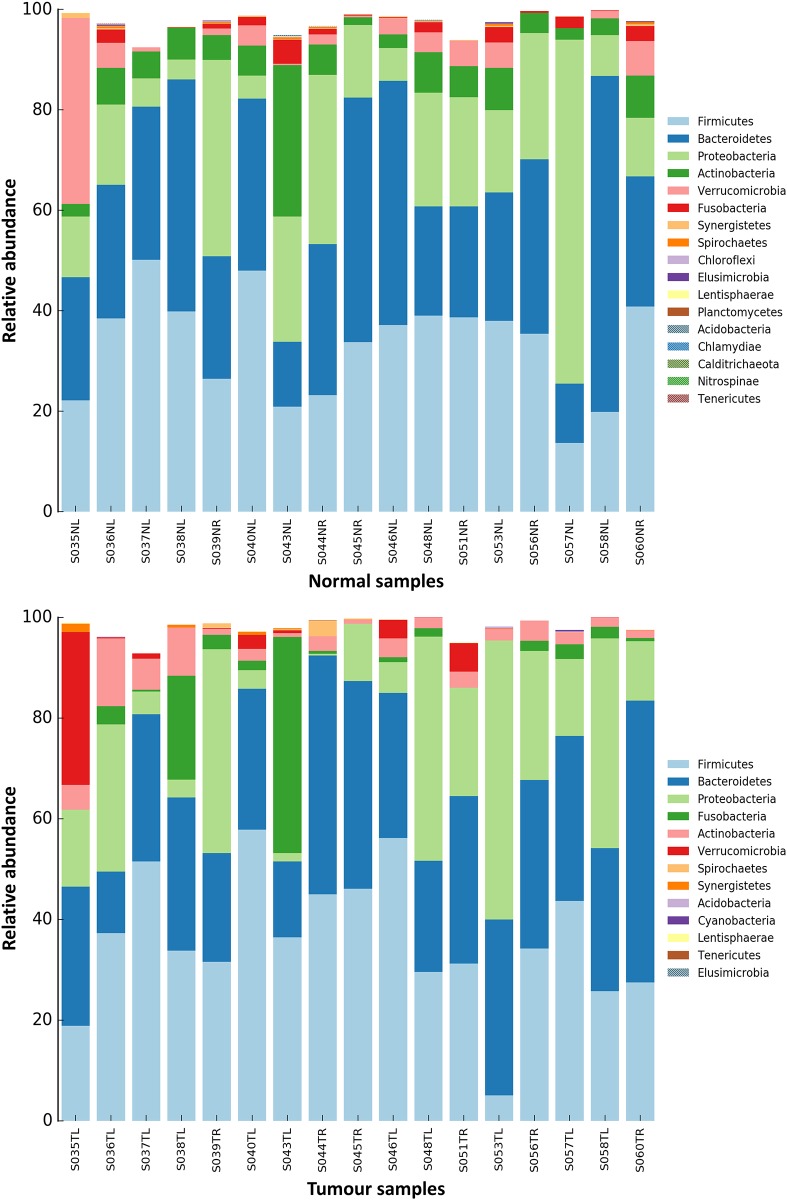
Bacterial phyla composition in normal and tumor samples.

In both tumor and normal tissues, Firmicutes, Bacteroidetes and Proteobacteria constituted the three most predominant phyla, at 36%, 30.7% and 19.5% of relative abundances, respectively, in the former samples, whilst the latter showed 33.3%, 31.5% and 19.7% of abundances (S3 Table). In the tumor samples, several bacterial phyla were shown to be nearly or completely absent including Calditrichaeota, Chlamydiae, Chloroflexi, Elusimicrobia and Planctomycetes (Fig 1).

### Alpha and beta diversity

**Fig 2:**
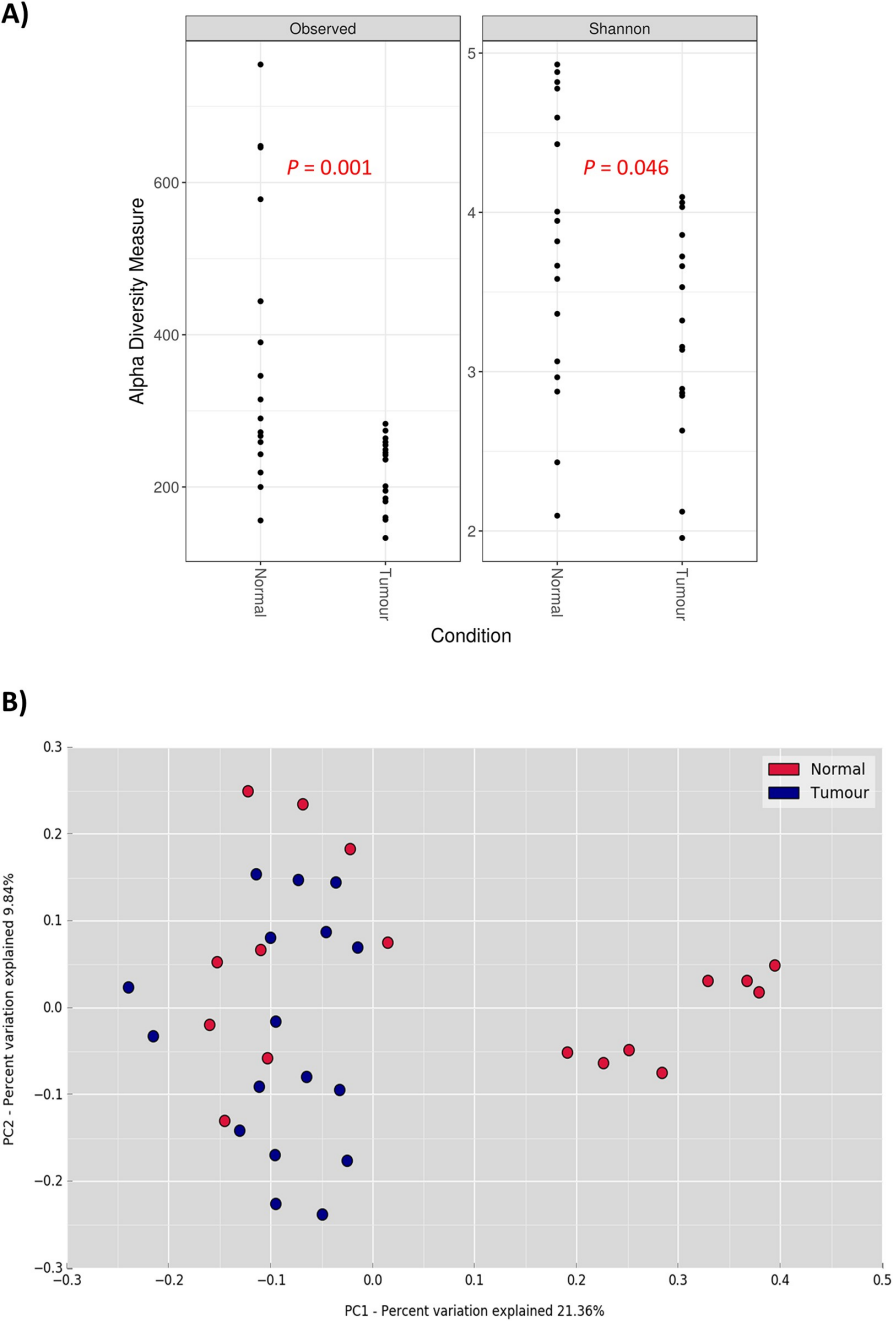
(A) Comparison of alpha diversity between normal and tumor samples based on species richness and Shannon diversity indexes. (B) PCoA plot of unweighted UniFrac distance of normal and tumor samples. Statistical testing using ANOSIM method revealed significant separation between normal and tumor samples (p-value = 0.003, R = 0.18).

To estimate and compare the alpha diversity of the colorectal microbial community derived from both tumor samples and normal tissue counterparts, we employed observed species and Shannon diversity indexes. The tumor samples had significantly reduced species richness and microbial diversity than the matched normal samples (observed species, *p*-value = 0.001; Shannon diversity, *p*-value = 0.046) (Fig 2A). To assess the overall difference of bacterial community between tumor and normal samples, PCoA based on unweighted UniFrac distance was performed. Two significantly distinct clusters were revealed (*p*-value = 0.003, R = 0.18) (Fig 2B). Eight normal samples were confined in one cluster and only two of these samples had been reported previously to have biofilm. Another cluster, interestingly, contained all tumor samples and the remaining normal samples [6]. The unweighted UniFrac distance PCoA indicates that histological condition of the samples has a more significant impact on the clustering than biofilm status. These normal samples that shared similar bacterial composition with the tumor samples could be a possible early indication of carcinogenesis, which merits further investigation.

### Taxonomic differences between tumor and paired normal tissue samples

**Fig 3:**
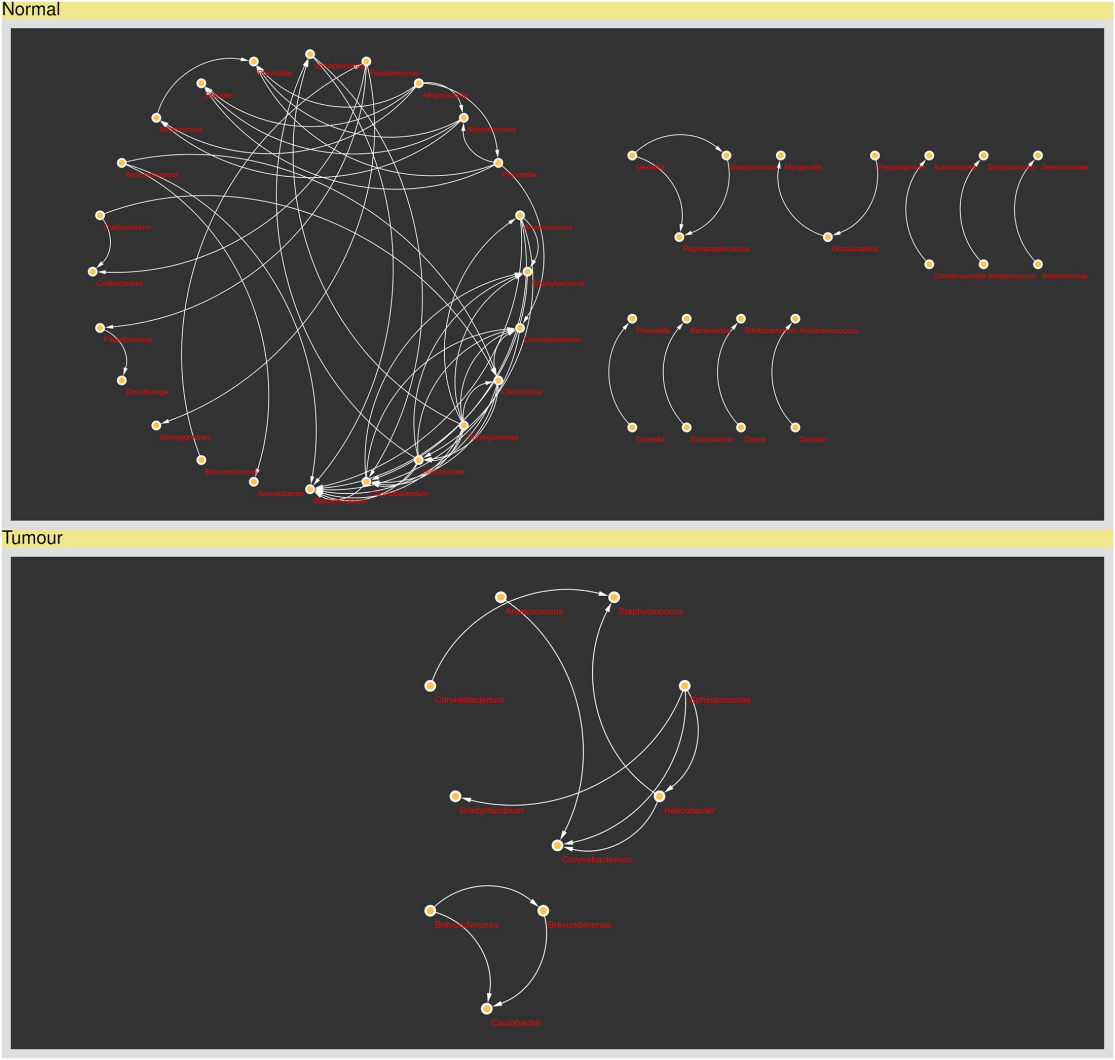
Co-occurrence network analysis of genus-level OTUs. Nodes corresponds to bacterial genera while edges represent positive correlations of at least 0.7 and with p-values less than 0.05. No negative correlations could be identified in this network analysis.

To identify bacterial genera that were significantly different between tumor and normal samples, genus-level OTUs that were present in at least 25% of total samples and with a minimum relative abundance of 0.001% were evaluated using Wilcoxon signed rank test. Of 358 OTUs tested, 24 were significantly enriched in normal tissue samples in comparison to their respective tumor counterparts (Table 5). These 24 OTUs represented 21 bacterial genera, in which *Alistipes* (median, 1.894 versus 0.024), *Oscillibacter* (median, 1.234 versus 0.009), *Bacteroides* (median, 0.847 versus 0.097), *Pseudoflavonifractor* (median, 0.012 versus 0) and *Succinivibrio* (median, 0.23 versus 0) being identified as the top five most differentially abundant genera. In addition, *Christensenella*, *Dialister and Pseudomonas* were assigned to two different OTUs, respectively. To examine if bacterial genera significantly affected by tissue status would deliver an impact on microbial interactions, we performed a co-occurrence network analysis based on Kendall’s tau correlation coefficient. As depicted in Fig 3, the density of interactions between different bacterial genera was profoundly less in the tumor samples as compared with the microbial interactions observed in the normal tissue counterparts. This is consistent with the abundances of several bacterial genera that were significantly decreased in the tumor samples such as *Succinivibrio*, *Bacteroides*, *Christensenella*, *Pseudomonas*, *Bifidobacterium*, *Dialister*, *Prevotella* and *Actinomyces*.

**Table 5:** List of genus-level OTUs that significantly differed between tumor samples and paired normal tissues by Wilcoxon signed rank testing.

OTU	Genus	Mean normal (%)	Mean tumor (%)	Median normal (%)	Median tumor (%)	*p*-value
**DENOVO80**	*Actinomyces*	0.341	0.066	0	0	0.009
**DENOVO7**	*Alistipes*	3.141	0.396	1.894	0.024	0.001
**DENOVO105**	*Anaerotruncus*	0.272	0.1	0.004	0	0.007
**DENOVO9**	*Bacteroides*	2.733	0.343	0.847	0.097	0.002
**DENOVO468**	*Bifidobacterium*	0.014	0	0.001	0	0.005
**DENOVO171**	*Christensenella*	0.056	0.028	0.015	0	0.004
**DENOVO74**	*Christensenella*	0.344	0.156	0.151	0	0.006
**DENOVO23**	*Collinsella*	1.282	0.644	1.108	0.307	0.007
**DENOVO73**	*Desulfovibrio*	0.414	0.204	0.256	0	0.003
**DENOVO244**	*Dialister*	0.029	0	0.007	0	0.005
**DENOVO48**	*Dialister*	0.743	0.35	0.037	0	0.008
**DENOVO200**	*Intestinimonas*	0.061	0.03	0.026	0	0.006
**DENOVO25**	*Megasphaera*	1.401	0.432	0.216	0	0.003
**DENOVO153**	*Mitsuokella*	0.094	0.026	0.001	0	0.008
**DENOVO85**	*Morganella*	0.315	0.033	0.116	0	0.008
**DENOVO352**	*Negativibacillus*	0.041	0.029	0.021	0	0.008
**DENOVO14**	*Oscillibacter*	2.085	1.027	1.234	0.09	0.001
**DENOVO732**	*Parabacteroides*	0.012	0.001	0.003	0	0.004
**DENOVO5**	*Prevotella*	5.036	2.734	1.285	0.066	0.009
**DENOVO611**	*Pseudoflavonifractor*	0.018	0.007	0.012	0	0.002
**DENOVO404**	*Pseudomonas*	0.082	4.07 × 10^−4^	0.022	0	0.004
**DENOVO20**	*Pseudomonas*	1.352	0.025	0.112	0.003	0.006
**DENOVO113**	*Rhodococcus*	0.132	0.001	0.004	0	0.006
**DENOVO31**	*Succinivibrio*	1.447	0.699	0.23	0	0.002

### Comparative functional differences of colorectal bacterial communities in tumor and paired normal tissue samples

We next employed Piphillin for the functional prediction of colorectal microbial communities between tumor and normal tissue samples, revealing 286 KEGG pathways. Eight KEGG pathways exhibited significant differences between both groups by Wilcoxon signed rank testing (*p*-value < 0.01, Table 6). The microbial communities of tumor samples had significant enrichments in both fatty acid biosynthesis and glycerolipid metabolism, while the normal microbial subsets were significantly enriched for pathways associated with citrate cycle, steroid biosynthesis, C5-branched dibasic acid metabolism, pantothenate and CoA biosynthesis, and sesquiterpenoid and triterpenoid biosynthesis.

**Table 6:** List of KEGG pathways that significantly differed between tumor samples and paired normal tissues by Wilcoxon signed rank testing.

KO identifier	Pathway description	Mean normal (%)	Mean tumor (%)	Median normal (%)	Median tumor (%)	*p*-value
**ko00020**	Citrate cycle (TCA cycle)	0.627	0.571	0.619	0.592	0.009
**ko00061**	Fatty acid biosynthesis	0.447	0.477	0.456	0.485	0.002
**ko00100**	Steroid biosynthesis	0.004	0.002	0.002	9.77 × 10^−5^	0.002
**ko00561**	Glycerolipid metabolism	0.257	0.279	0.258	0.273	0.004
**ko00660**	C5-Branched dibasic acid metabolism	0.267	0.254	0.27	0.255	0.009
**ko00770**	Pantothenate and CoA biosynthesis	0.539	0.519	0.536	0.504	0.005
**ko00909**	Sesquiterpenoid and triterpenoid biosynthesis	0.009	0.005	0.005	3.02 × 10^−4^	0.001
**Ko04976**	Bile secretion	<0.001	<0.001	<0.001	<0.001	0.008
**ko05131**	Shigellosis	0.001	4.79 × 10^−4^	0.001	4.73 × 10^−5^	0.002

**Fig 4:**
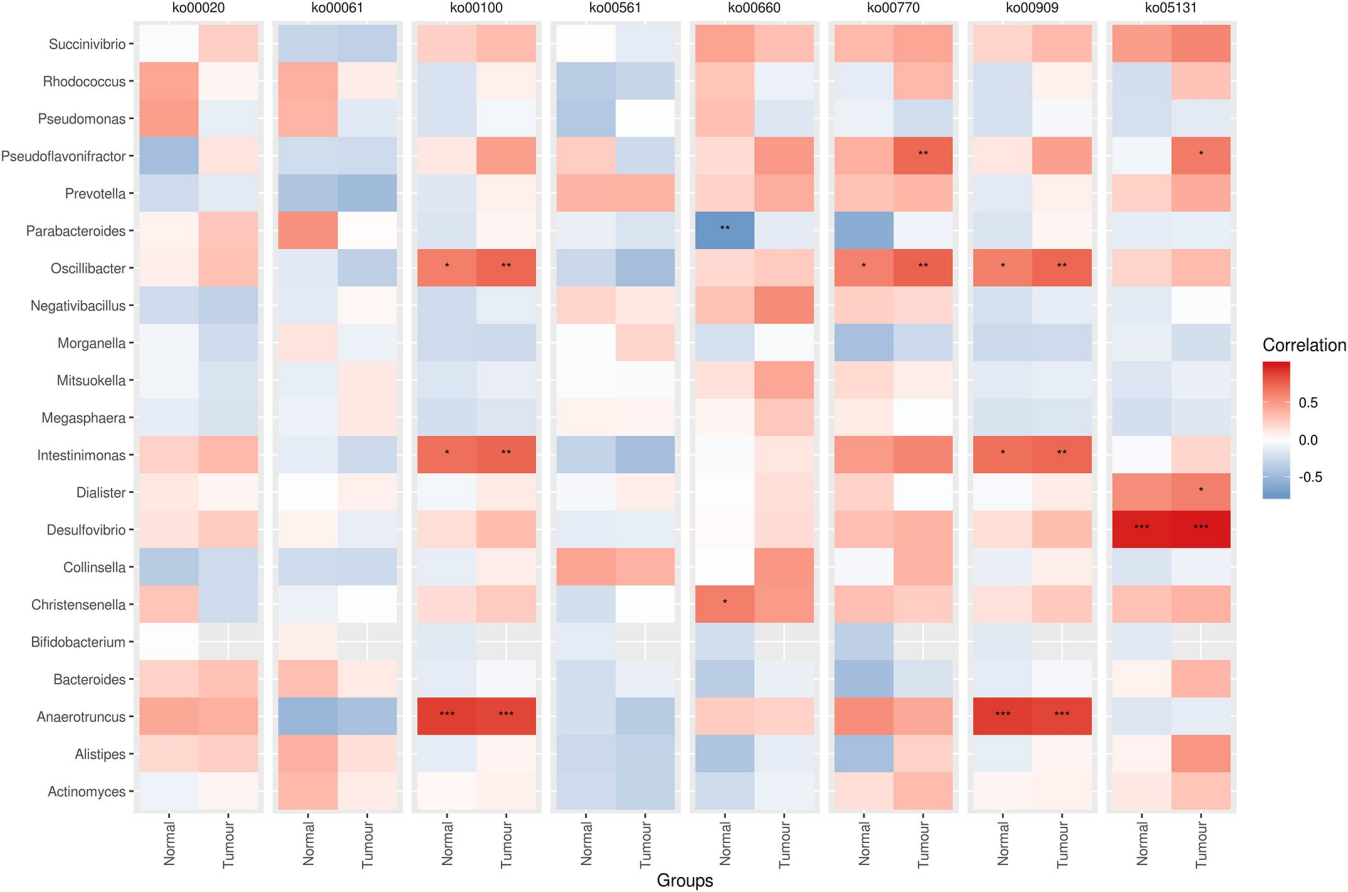
Pearson’s correlation between bacterial genera and KEGG pathways with significant differences. ko00020: Citrate cycle (TCA cycle); ko00061: Fatty acid biosynthesis; ko00100: Steroid biosynthesis; ko00561: Glycerolipid metabolism; ko00660: C5-Branched dibasic acid metabolism; ko00770: Pantothenate and CoA biosynthesis; ko00909: Sesquiterpenoid and triterpenoid biosynthesis; ko05131: Shigellosis.

Pearson’s correlation analysis of bacterial genera and KEGG pathways that significantly differed between the tumor and normal tissue samples revealed several significant associations. The abundances of *Anaerotruncus*, *Intestinimonas* and *Oscillibacter* exhibited significant relationships with both steroid biosynthesis and sesquiterpenoid and triterpenoid biosynthesis pathways in both tumor and normal tissue samples with *Anaerotruncus* showing the most significant associations (*p*-values < 0.0001) (Fig 4; S4 Table). *Oscillibacter* was also significantly associated with pantothenate and CoA biosynthesis (*p*-value < 0.01, normal group; *p*-value < 0.001, tumor group). The C5-branched dibasic acid metabolism pathway in the normal group was positively associated with *Christensenella* and inversely correlated to *Parabacteroides*, with *p-values* < 0.01 and < 0.001, respectively. The shigellosis pathway that significantly differed between both tumor and normal samples was strongly correlated to the abundance of *Desulfovibrio* bacteria in each group, respectively (*p*-values < 0.0001).

**Fig 5:**
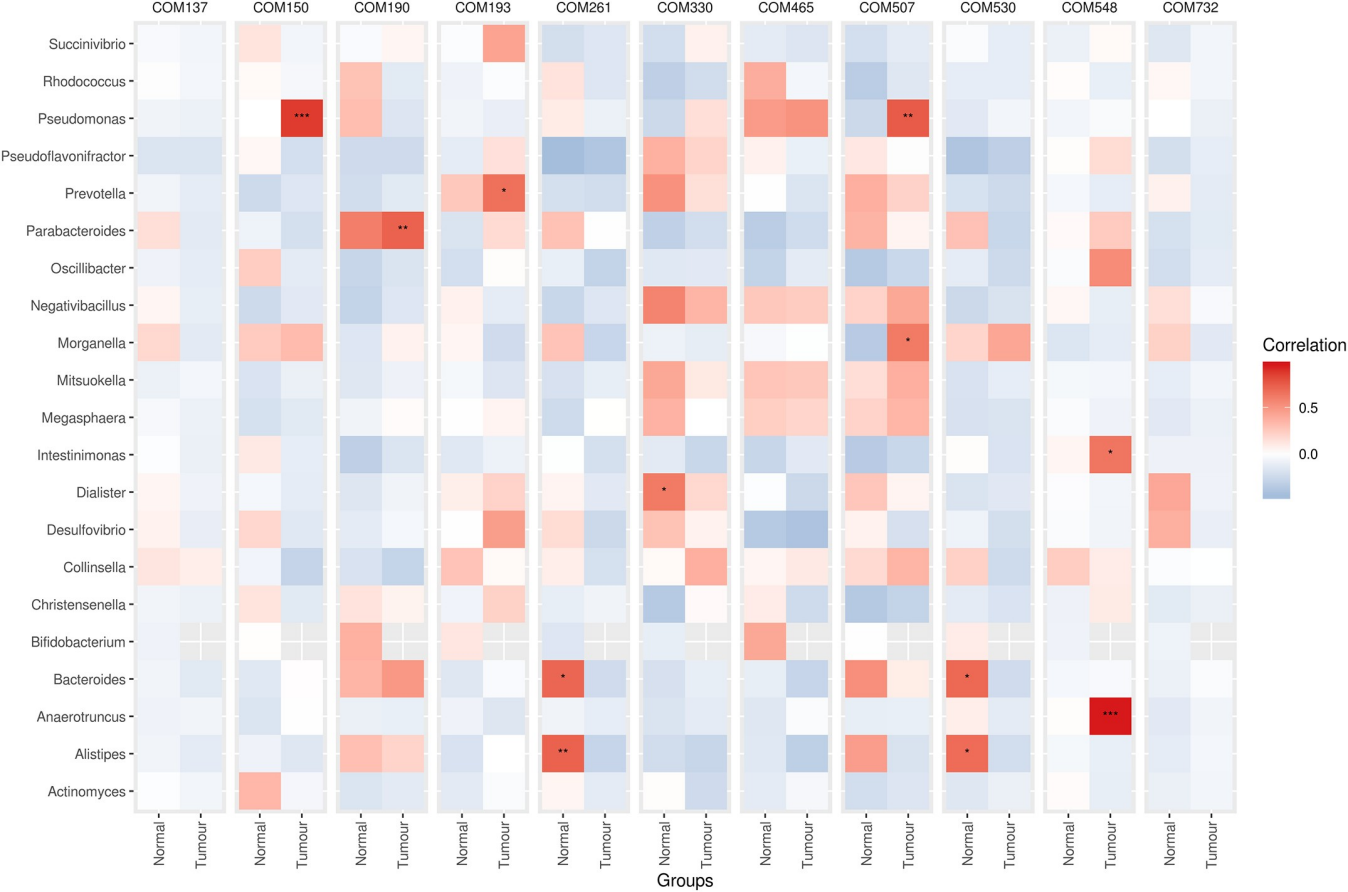
Pearson’s correlation between bacterial genera and metabolites with significant differences. COM137, 5α-Androstan-3β-ol-17-one sulfate; COM150: 6-Methoxyquinoline; COM190: Antillatoxin B; COM193: Arg Arg Met; COM261: Creatine; COM330: Formylmethionyl-leucyl-phenylalanine methyl ester; COM465: m-Coumaric acid; COM507: N(alpha)-t-Butoxycarbonyl-L-leucine; COM530: PA(18:4(6Z,9Z,12Z,15Z)/20:4(5Z,8Z,11Z,14Z)); COM548: PE(P-16:0/0:0); COM732: Val Arg Phe.

Pearson’s correlation analysis of metabolites and bacterial genera that significantly differed between the tumor and normal tissue samples also revealed several significant associations. In the tumor group, PE(P-16:0/0:0) exhibited significant relationships with *Anaerotruncus* (*p*-value < 0.0001) and *Intestinimonas* (*p*-value < 0.01) (Fig 5; S5 Table). *Pseudomona* correlated significantly with 6-methoxyquinoline (*p*-value < 0.0001) and N(alpha)-t-butoxycarbonyl-L-leucine (*p*-value < 0.001), which also correlated significantly with *Morganella* (*p*-value < 0.01). Parabacteroides and Prevotella correlated significantly with Antillatoxin B (*p*-value < 0.001) and Arg Arg Met (*p*-value < 0.01) respectively. On the other hand, in the normal samples, *Alistipes* and *Bacteroides* showed significantly association with both creatine (*p*-value < 0.001; 0.01) and PA(18:4(6Z,9Z,12Z,15Z)/20:4(5Z,8Z,11Z,14Z)) (*p*-value < 0.01). *Dialister* was also found to be significantly associated with formylmethionyl-leucyl-phenylalanine methyl ester (*p*-value < 0.01).

## Discussion

Metabolomics enables a large-scale, qualitative, and quantitative study of metabolites in a system biological approach. Unlike mRNAs and proteins, biosynthesis of metabolites is complex and requires advanced instrumentations such as MS, NMR spectroscopy, and laser-stimulated fluorescence (LSF) spectroscopy. Notably, each of these instruments has their unique strengths and limitations. Although NMR is highly selective and non-destructive and is the gold standard in metabolite structural elucidation, it has relatively lower sensitivity compared to other technologies [24]. In contrast, LSF is one of the most sensitive techniques, but it is not chemically selectivity and this limits its usefulness in structural identification of metabolites in complex biological systems [24]. On the other hand, MS, which provides good balance of sensitivity and selectivity, is frequently used in metabolomic analyses of complex biological samples [24]. Coupling chromatography to MS provides a great resolution for metabolomics identification and quantification. Currently, GC, LC, and capillary electrophoresis (CE) have been incorporated into MS-based metabolomics. GC-MS, which is suitable for the analysis of volatile, thermally stable, and energetically stable compounds, is extensively used for routine primary metabolite studies of common but important metabolite classes such as amino acids, organic acids and free fatty acids. CE-MS is inherently low in sensitivity, poor in reproducibility, and may be affected by electrochemical reactions of metabolites. Recently, Büscher et al. compared the performances of GC-MS, LC-MS, and CE-MS in application to quantitative metabolomics, and demonstrated that CE-MS was the least effective platform for analyzing complex biological samples [25]. Thus, LC-MS was chosen in this study for discovering unknown metabolites by untargeted metabolomics based on the wider range of compounds it can analyze.

Tian et al. analyzed the metabolomic signatures of CRC tissues and their adjacent non-involved tissues from Chinese patients using high-resolution magic-angle spinning (HRMAS) 1H NMR spectroscopy in combination with GC-FID/MS [26]. In that study, tissue metabolic phenotypes (in energy metabolism, membrane biosynthesis and degradations, osmotic regulation, and proteins and nucleotides metabolism) was able to discriminated CRC tissues from adjacent non-involved tissues [26]. More recently, Satoh et al., using CE-MS metabolome for profiling paired tumor and normal tissue from Japanese patients with CRC, found that S-adenosylmethionine (SAM) was the most up-regulated metabolite in tumor tissue [27]. The LC-MS-based metabolomics approach of this study provides additional information that complements our current understanding of the metabolomic differences between CRC tissues and adjacent non-involved tissues. In this study, it was shown that diverse metabolic pathways (such as N-glycan biosynthesis, carotenoid biosynthesis, cholesterol metabolism, bile acid metabolism, pentose and glucuronate interconversions, biosynthesis of secondary metabolites, amino acid metabolism and steroid hormone biosynthesis) differs between tumor and normal tissues. Consistent with previous studies [26,27], molecular evidence from this study suggests that cancer cells may alter their metabolism for the production of macromolecular precursors in CRC. The finding of SAM by Satoh et al. and subsequently SAH in our study to be frequently elevated in tumor tissues highlighted the importance of cysteine and methionine metabolism in carcinogenesis. Methionine, an essential amino acid in protein synthesis, is the precursor to SAM required by a variety of methyltransferases for the methylation of DNA, RNA, proteins, and lipids [28]. When SAM releases activated methyl group in methylation reactions, it is transformed into SAH that is further hydrolyzed to homocysteine [28]. Sibani et al. has demonstrated that there was positive correlations between SAM, SAH, and DNA hypomethylation with cellular transformation under folate-adequate conditions in pre-neoplastic small intestine of multiple intestinal neoplasia (Min) mice [29], thus illustrating the importance of SAM and SAH in DNA methylation and colorectal carcinogenesis. Furthermore, several cancer cells utilize SAM for hyperactive polyamine synthesis [28]. In turn, polyamine putrescine reacts with a decarboxylated form of SAM to form spermidine and spermine [28]. Therefore, this may explain the elevation of SAH and depletion of spermidine at tumor sites compared surrounding non-affected mucosa (Table 3).

Serotonin, 5-hydroxytryptamine (5-HT) is mainly synthesized at the gastrointestinal (GI) tract and it is closely associated with GI function and physiology as extensively reviewed by Manocha and Khan [30]. In intestinal enterochromaffin (EC) cells, conversion of dietary tryptophan is the first step in the biosynthesis of serotonin, has been implicated in various GI diseases and functional disorders [30]. Alteration in serotonin signaling is associated with celiac disease, CRC, and diverticular disease [30]. The absence of serotonin at tumor sites compared to corresponding adjacent non-involved sites may suggest increased catabolism of serotonin by cancerous cells. Serotonin is essential for the growth of s.c. colon cancer allografts *in vivo* by acting as a regulator of angiogenesis which reduces the expression of matrix metalloproteinase 12 (MMP-12)—an endogenous inhibitor of angiogenesis—in tumor-infiltrating macrophages [31]. The intricate interactions of the gut microbiota, food consumed, and intestinal cells together will impact the serotonin production, secretion, and degradation, and, hence, may be accountable for the impaired function of serotonin in GI diseases [30]. Thus, modulation of tryptophan metabolism, such as the production of serotonin can be used as a potential therapeutic strategy for CRC in the future.

2,2'-Diketospirilloxanthin is a naturally occurring carotenoid. Carotenoids are organic pigments produced by many plants and algae, as well as by various bacteria and fungi [32]. Natural carotenoids, because of their antioxidant properties, have been suggested to have anticarcinogenic activity [32]. On the other hand, a prospective Multiethnic Cohort Study based on quantitative food frequency questionnaires did not find any significant association between intake of individual and total carotenoids and CRC risk [33]. The detection of 2,2'-diketospirilloxanthin at cancerous sites but not adjacent non-involved sites suggests that the carotenoid may have local protective effects on epithelial tissues.

The detection of metabolites involved in antibiotics biosynthesis in CRC tissues suggests a role of microbiota structure and composition in colorectal carcinogenesis. Supported by data from meta-omics analyses and mechanistic studies *in vitro* and *in vivo*, bacteria, such as *Fusobacterium nucleatum*, enterotoxigenic *Bacteroides fragilis*, and colibactin-producing *Escherichia coli*, may be potentiators for CRC development [34]. In addition, it has been demonstrated that functional predictions from *16S rRNA* gene sequences and metabolomics support that colonic mucosal biofilm contributes to antibiotic biosynthesis leading to alteration of the cancer metabolome to regulate cellular proliferation and colon cancer growth potentially affecting cancer development and progression [6,10,35]. The study of microbiome differences between tumor and surrounding non-affected tissues in this study has highlighted both taxonomical and predicted functional differences between normal and cancerous tissues. Predicted functions implied from the *16S rRNA* gene sequences are consistent with findings from metabolomics analysis showing depletion of bacteria genera involve in steroid biosynthesis, terpenoid biosynthesis, and bile secretion in tumor relative to paired normal tissues. In addition, significant correlation was found between *Anaerotruncus*, *Intestinimonas* and *Oscillibacter* and steroid and terpenoid biosyntheses. However, the abundance of bacteria genera associated with bile secretion pathway was low (<0.001%). Nevertheless, the human host is known to produce large, conjugated and hydrophilic bile acids. Members of the intestinal microbiome may utilize bile acids and their conjugates resulting in smaller, unconjugated and hydrophobic bile acids. These unconjugated bile acids induce oncogenesis in colonic epithelial cells by altering muscarinic 3 receptor (M3R) and Wnt/ β-catenin signaling and thus act potential promoters of colon cancer [36]. Interestingly, many naturally occurring triterpenoids have been shown to exhibit cytotoxicity against tumor cells, as well as demonstrated to have anticancer efficacy *in vivo* [37].

In conclusion, this study expanded our insight into localized metabolic and microbiome differences between tumor and normal colonic tissues in CRC patients. Besides providing deeper understanding of the pathogenic process of colorectal carcinogenesis, these functional metabolites have potential implications in both the drug discovery process and in precision medicine. Future large-scale meta-analysis could be carried out by comparing the current and other datasets collected from different parts of the world to explore the association between the geographical factors with the metabolic differences in the CRC patients.

## Supporting information

S1 Table List of compounds identified by LCMS. (XLSX)

S2 Table Summary statistics of sequencing data. (XLSX)

S3 Table Taxonomic composition at phylum level. (XLSX)

S4 Table Pearson’s correlation between bacterial genera and KEGG pathways. (XLSX)

S5 Table Pearson’s correlation between bacterial genera and metabolites. (XLSX)
